# Long non-coding RNAs in glial cells: key drivers of neuroinflammation in cognitive disorders

**DOI:** 10.3389/fnagi.2026.1849634

**Published:** 2026-07-31

**Authors:** Chiara Malatino, Ivana Raffaele, Angelo Quartarone, Gabriele Raciti, Ivan Anchesi, Nunzio Iraci, Maria Cristina De Cola

**Affiliations:** 1 Policlinico Universitario G. Martino di Messina – UOC Patologia Clinica, Messina, Italy; 2 IRCCS Centro Neurolesi "Bonino-Pulejo", Via Provinciale Palermo, Contrada Casazza, Messina, Italy; 3 Department of Biomedical and Biotechnological Sciences, University of Catania, Catania, Italy

**Keywords:** cognitive disorders, glia, long non-coding RNAs, neurodegenerative diseases, neuroinflammation, neuroprotection

## Abstract

Neurodegenerative diseases (NDs) are characterized by the progressive deterioration of cognitive and motor functions. In this context, glial cell-mediated neuroinflammation is recognized as a key driver of disease progression. Long non-coding RNAs (lncRNAs) have emerged as key epigenetic regulators that modulate gene expression and inflammatory signaling pathways in this context. Due to their high cell-type specificity and dynamic regulation, lncRNAs are promising diagnostic biomarkers and therapeutic targets for NDs. The balance between the neuroprotective and proinflammatory functions of glial cells plays a crucial role in ND progression. LncRNAs act as multifunctional modulators of glial activity, influencing neuroinflammatory responses, astrocyte and microglia dysfunction, and the clearance of toxic protein aggregates. Several lncRNAs, including RMST, MALAT1, and NEAT1, regulate inflammatory pathways through various molecular mechanisms. For example, they act as competing endogenous RNAs that absorb microRNAs. These regulatory networks influence key signaling cascades involved in neuroinflammation, including Toll-like receptor (TLR)-mediated pathways, the NF-κB signaling axis, and NLRP3 inflammasome activation. In this review, we summarize and categorize glial lncRNAs according to their molecular interactions and functional roles in disorders related to cognitive decline. By integrating current evidence, we highlight the contribution of lncRNA-mediated regulatory networks to neuroinflammatory processes and discuss their potential as biomarkers and therapeutic targets. Our findings suggest that glial lncRNAs are crucial regulators of neuroinflammation in cognitive disorders. Their ability to modulate pathways such as the NLRP3 inflammasome makes them promising diagnostic biomarkers and therapeutic targets. Targeting these molecular networks provides new opportunities to halt neurodegeneration and improve clinical outcomes.

## 1 Introduction

Neurodegenerative diseases (NDs) are characterized by progressive and irreversible deterioration of the function of brain cells, resulting in functional alterations of the Central Nervous System (CNS) such as cognitive, movement and memory problems (García-Fonseca et al., 2021; Jiang et al., 2022). NDs significantly compromise the quality of life of patients and their caregivers, resulting in heavy economic and social burdens (De Cola et al., 2017). Therefore, it is essential and urgent to find new treatments that not only address the symptoms but also significantly delay the progression of the disease. Unfortunately, the specificity of the diagnosis of NDs is very poor due to a large clinical variability in the presentation of early symptoms of the diseases (Hou et al., 2019; Villain and Planche, 2024). Among the processes involved in cognitive impairment, neuroinflammation is believed to be a key component of NDs. It is sustained by the dysregulated release of a plethora of molecules (e.g., cytokines, chemokines etc.) mediated by glial cells (Zhang et al., 2023). Glial cells, which include astrocytes, oligodendrocytes, and microglia, are active players in the CNS. Their role in orchestrating function goes far beyond structural support, including the active modulation of synaptic transmission, metabolic support, and regulation of nerve conduction velocity. This dynamic function, crucial for development and remodeling of neural circuits, is fundamental to brain homeostasis and plasticity (Allen and Lyons, 2018). In NDs, microglia play a dual role: protective, clearing debris and restoring homeostasis, and harmful, promoting neuroinflammation and synaptic loss. This toxic microglial activity also triggers a reactivity in astrocytes, which transform from supportive cells into neurotoxic ones, creating a vicious cycle that accelerates neuronal damage (Gao et al., 2023; Patani et al., 2023). Thus, their excessive and prolonged activation contributes to neurodegeneration and increases protein aggregation (Zhang et al., 2023). The process of glial dysregulation, which leads to chronic and damaging activation, is orchestrated by complex molecular mechanisms (Singh et al., 2020).

In this context, lncRNAs—transcript exceeding 200 nucleotides that lack protein-coding potential—have emerged as pivotal epigenetic and transcriptional modulators, controlling susceptibility genes involved in neurological disorders (Aliperti et al., 2021; Singh et al., 2025). Due to their high cell-type specificity and direct involvement in both homeostatic and pathological states, lncRNAs represent promising diagnostic biomarkers and innovative therapeutic targets for neuroinflammation and cognitive disorders (Zeng et al., 2023). In particular, targeting lncRNAs within protein aggregates presents a novel therapeutic avenue for these otherwise intractable conditions, owing to their involvement in co-regulatory pathways across different NDs (Xu et al., 2025). This approach underscores their viability not only as distinctive biomarkers but also as promising therapeutic targets. In this review, we focused on identifying lncRNAs enriched in glial population—specifically those dysregulated in conditions of astrocytosis or microgliosis—in affecting neurodegeneration and neuroinflammation, through complex regulatory networks. These glial responses, in fact, are hallmark features of cognitive neurodegenerative disorders, acting as primary drivers of disease progression. Consequently, a new therapeutic strategy may leverage the targeted modulation of these specific glial lncRNAs to counteract cognitive decline.

## 2 Glial cells

Recent evidence suggests that chronic neuroinflammation not only promotes progression but may also be an important initiator of neurodegeneration, thereby accelerating the cognitive decline processes (Mekhora et al., 2024). Progressive proteinopathies, including Alzheimer’s disease (AD), Parkinson’s disease (PD), and amyotrophic lateral sclerosis, are characterized by the accumulation of misfolded proteins in specific areas of the brain and are linked to inflammatory processes and altered glial activity (Bernaus et al., 2020). For many years, glial cells were considered to be merely a “scaffold” for neurons. However, it is now well established that they are a significant and active components of the CNS (Carter et al., 2019). The different types of glial cell are astrocytes, microglia, oligodendrocytes and oligodendrocyte precursor cells (OPCs). These cell types play a crucial role during development, in response to injury, and in maintaining the balance of nervous tissue (Jessen, 2004). The behavior of glial cells depends on complex intercellular communication with neurons, astrocytes, the Blood–Brain Barrier (BBB), and T cells within the CNS. Importantly, this crosstalk is activated by specific triggers unique to each neurological condition. Clinical trials show that anti-inflammatory therapies reduce aggregates but fail to stop disease progression. This reflects the complexity of inflammatory signals, which often have both beneficial and harmful roles depending on the stage of the disease (Zhang et al., 2023). Astrocytes are named by their characteristic star-like shape, which is made up of a small cell body from which extend numerous swelling and shrinking thin processes (Gao and Hu, 2019). In addition to providing structural and metabolic support to neurons, they regulate synaptic transmission and blood flow in the brain. Indeed, astrocytes play a pivotal role in neuronal metabolism, synapse maintenance, neurotransmitter recycling to prevent excitotoxicity, and the maintenance of the BBB. Astrocytes undergo molecular and functional changes through a process called “reactive astrogliosis” in response to endogenous or exogenous damage. If the reactive state persists, astrocytes release excessive and uncontrolled amounts of inflammatory factors, which lead to the remodeling of neuronal tissue and the development of brain pathology (Schröder et al., 2024). Furthermore, it is well known that the misfolding and intracellular and extracellular accumulation of protein aggregates are common features of NDs. The proteinopathies that have been most extensively studied involve *β*-amyloid (Aβ), tau and alpha-synuclein (*α*-Syn). While microglia predominantly remove protein aggregates, astrocytes appear to contribute to this process through pinocytosis or phagocytosis (Giusti et al., 2024). They have also been shown to play a significant role in the removal of Aβ, both through endocytosis and through the production of enzymes capable of degrading protein aggregates (Carter et al., 2019; Wang et al., 2024). Microglial cells are macrophages that reside in the brain parenchyma, interacting closely with astrocytes and neurons, and constantly monitoring the extracellular environment (Colonna and Butovsky, 2017). These cells possess receptors that enable them to respond to a wide range of signals that alter tissue homeostasis. Following a brain event, microglia undergo massive activation, known as ‘reactive microgliosis’: the cells take on an amoeboid morphology and release inflammatory factors that can limit or exacerbate neuronal damage (Aguzzi et al., 2013). In fact, recent single-cell transcriptomics and metabolomics studies have revealed microglial subtypes with distinct transcriptional and metabolic profiles, representing a dynamic continuum of functional states that vary depending on the microenvironment, stage, and type of disease (Lu et al., 2025). A very interesting and recent study by Baek et al. (2022) has highlighted the presence of two distinct functional networks in human microglia cells activated by LPS stimulation, thereby demonstrating that microglia can simultaneously activate defense pathways and other potentially cytotoxic pathways under the epigenetic control of lncRNAs. Unlike astrocytes and microglia, OPC and oligodendrocyte cell lines are relatively less well known, but recent evidence highlights the role of OPCs as surveillance cells of the brain parenchyma and that of oligodendrocytes as key cells in the regulation and development of neural synapses and synaptic plasticity (Liu et al., 2023).

There is a growing awareness of the role of glial cells in NDs. Their hyperactivation results in the massive release of neurotoxic factors, causing neuronal damage (Afridi et al., 2022). However, the factors that cause glial cells to switch from a neuroprotective to a neuroinflammatory role are not yet fully understood (Yang et al., 2023). Nevertheless, it can be argued that these cells not only respond to neurodegeneration, but can also act as true pathogenic drivers. In this regard, it should be noted that atrophic and reactive astrocytes have been identified in the post-mortem brain tissue of patients with AD and in animal models of AD (Schröder et al., 2024). In addition to histological evidence, genome-wide association studies (GWAS) and post-GWAS studies have identified genetic variants that directly influence glial function in familial forms of AD and PD (Yang et al., 2023; Brandebura et al., 2023).

## 3 LncRNAs: key regulators in physiology, pathology, and cell fate

The specific biogenesis and processing of lncRNAs are primary determinants of their subcellular compartmentalization and, by extension, their functional specializations (Statello et al., 2021). A significant repertoire is selectively retained within the nucleus to orchestrate chromatin architecture, DNA methylation, and transcription factor activity, in various biological processes, including development, cell differentiation, and disease response (Quinn and Chang, 2016). Alternatively, certain lncRNAs are exported to the cytoplasm to modulate messenger RNAs (mRNAs), microRNAs (miRNAs), and circular RNAs (circRNAs), thereby fine-tuning complex regulatory networks. Furthermore, specific lncRNAs are packaged into exosomes and secreted into biological fluids, including blood and cerebrospinal fluid. This extracellular transport facilitates systemic signaling and establishes these transcripts as robust, high-fidelity diagnostic biomarkers for NDs (Kuo et al., 2021). However, lncRNAs do not translate into proteins but their regulatory function is essential for the life of complex organisms (Dinger et al., 2008). Although transcribed predominantly by RNA polymerase II, lncRNAs can also originate from Pol I and III activity. These processes give rise to molecules often subject to capping, polyadenylation, and extensive alternative splicing, resulting in a diverse array of complex isoforms (Mattick et al., 2023). A large fraction of the genomes of complex organisms is occupied by the genes that specify lncRNAs. Researchers distinguish different types of molecules based on their genomic location and orientation relative to protein-coding genes (Uszczynska-Ratajczak et al., 2018). A lncRNA is defined as “sense” when it is transcribed from the same strand as a coding gene and partially or completely overlaps with it (Ma et al., 2013). It can overlap with exons, introns, or the untranslated regions (UTRs) of the coding gene (Wright et al., 2022). A lncRNA is defined as “antisense” when it is transcribed from the strand opposite the coding gene, giving rise to a complementary strand and partially or completely overlapping with the gene itself. Antisense lncRNAs represent one of the most abundant classes of lncRNAs and are often involved in the cis-regulation of nearby genes (Ponting et al., 2009; Chodurska and Kunej, 2025). LncRNAs come from various genomic regions, affecting their production and processing. They are often created from active enhancer regions, promoter regions, and pseudogenes (Chodurska and Kunej, 2025).

Functionally, lncRNAs interact with DNA, RNA, and proteins, acting as guides, decoys (or sponge), or scaffolds within epigenetic or transcriptional complexes. They can form triplex structures with DNA or interact with proteins and transcription factors, influencing enhancer activity, chromatin loop formation, and transcription regulation (Kuo et al., 2021; Ponting et al., 2009). LncRNAs act both in the nucleus and in the cytoplasm and can also be secreted in extracellular vesicles (EVs)—nanosized lipid particles—facilitating the exchange of epigenetic information between cells (Stewart et al., 2024; Marchetti et al., 2020). LncRNAs delivered by astrocyte-derived EVs have a key role in modulating neuroinflammation. Indeed, they provide neuroprotective effects, directly or indirectly, influencing the function of other glial cells and supporting brain homeostasis in response to toxic insults (Stewart et al., 2024; Leggio et al., 2022). LncRNAs can also act as inhibitors of miRNAs, which are small, single-stranded, ncRNA molecules involved in the post-transcriptional regulation of gene expression (Meng et al., 2021). Along with circRNAs, lncRNAs act by sequestering miRNAs, directly influencing their availability for mRNA binding and precisely tuning gene expression (Chen and Kim, 2024). This “sponge” activity creates an intricate competitive endogenous RNA (ceRNA) network, a fundamental post-transcriptional mechanism for cellular homeostasis that is often dysregulated in disease. In this regulatory model, lncRNAs and mRNAs compete for shared binding sites to achieve differential gene control; consequently, ceRNA networks identify complex crosstalk between different classes of RNAs through miRNA-binding sites (Gil-Jaramillo et al., 2023). Notably, lncRNAs are dynamically expressed during differentiation and development, responding to environmental stimuli, and are involved in numerous biological processes, such as cell proliferation and differentiation, transcription, post-transcriptional modifications, chromatin organization and cellular physiological and pathological processes (Jiang et al., 2022; Schröder et al., 2024; Ou et al., 2021). Also, they have a significant impact on the pathogenesis of numerous diseases, including NDs and cancer, acting as both oncogenes and tumor suppressors (Jiang et al., 2022; Li and Liu, 2019). Studies in the field of neuroinflammation suggest an important role for ncRNAs in the onset, development, and possibly even for the treatment of NDs. It is widely recognized that the detection of altered and specific ncRNA expression profiles for patient cohorts with particular NDs can represent a robust biomarker. This approach offers a standardized and minimally invasive alternative to improve diagnostic accuracy and monitor the clinical course of the disease (follow-up) (Gil-Jaramillo et al., 2023; Mosquera-Heredia et al., 2024; Luo et al., 2023). In fact, lncRNAs modulate the production of pro-inflammatory cytokines, playing a role as regulators of the innate immune response, thus influencing the activity of microglia (El Idrissi et al., 2021). Some of these lncRNAs are well characterized for their pro-inflammatory role, such as MIR155HG and 2500002B13Rik, often acting via the Nuclear Factor kappa-light-chain-enhancer of activated B cells (NF-κB) pathway. On the other hand, others are associated with protective phenotypes, such as Mir99ahg, linked to anti-inflammatory and differentiation processes (Kim et al., 2023).

**Figure 1:**
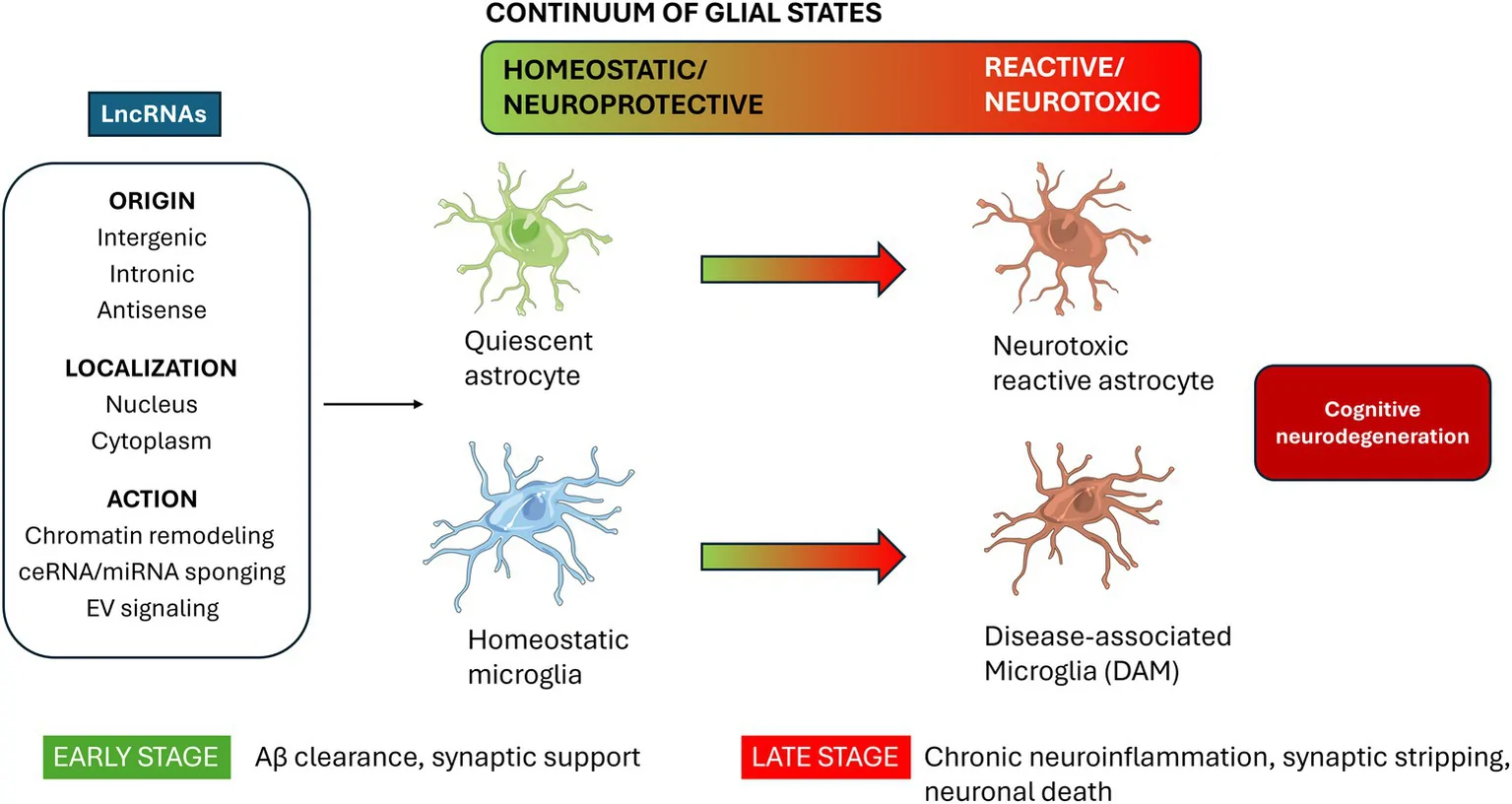
ncRNAs modulate glial cells along a continuum of functional states. Long non-coding RNAs (lncRNAs) arise from diverse genomic origins (intergenic, intronic, antisense), localize to both the nucleus and the cytoplasm, and act through chromatin remodeling, ceRNA/miRNA sponging, and extracellular vesicle (EV) signaling. Through these mechanisms they modulate the functional state of glial cells. Rather than switching between two fixed phenotypes, astrocytes and microglia occupy a dynamic continuum of glial states, ranging from a homeostatic/neuroprotective pole to a reactive/neurotoxic pole: Astrocytes shift from a quiescent to a neurotoxic reactive phenotype, and microglia from a homeostatic state to disease-associated microglia (DAM). This transition is stage-dependent: In the early stage, glial responses favor Aβ clearance and synaptic support, whereas in the late stage the persistence of reactive states drives chronic neuroinflammation, synaptic stripping, and neuronal death, ultimately promoting cognitive neurodegeneration. Cell illustrations were created using Servier Medical Art (https://smart.servier.com/); Servier Medical Art is licensed under CC BY 4.0 (https://creativecommons.org/licenses/by/4.0/). Aβ, amyloid-beta; ceRNA, competing endogenous RNA; DAM, disease-associated microglia; EV, extracellular vesicle; lncRNA, long non-coding RNA; miRNA, microRNA.

The identification of lncRNAs and their co-expression networks provides crucial clues to understanding the molecular mechanisms underlying microglial activation and its impact on NDs. A schematic overview summarizing the role of astrocyte- and microglia-lncRNAs in sustaining neuroinflammation and modulating neurotoxic or neuroprotective responses is presented in Figure 1.

## 4 Methods

### 4.1 Data sources and keywords

A systematic review in line with the Preferred Reporting Items for Systematic Reviews and Meta-Analyses (PRISMA) guidelines was performed. Articles published up to December 31, 2025, were searched on the PubMed (All fields), Web of Science (All fields), and Scopus (title, abstract, keywords) databases, without language restrictions. Databases were queried using key words and their combinations as in the following query: (“long non-coding RNA” OR “lncRNA”) AND (neurology OR neurodegenerative OR neurotrophic OR neurologic OR neuroinflammation) AND (glial OR astrocyte OR microglia OR gliosis OR astrogliosis OR microgliosis). To guarantee the inclusion of databases that do not use MeSH indexing, MeSH terms were not used. Therefore, the search strategy primarily relied on carefully selected free-text keywords to maximize sensitivity and ensure the inclusion of the broadest range of relevant studies. Only studies published in the last 10 years were selected because the investigation of glial cells in neurodegeneration is a rapidly evolving area of research. This time frame was chosen to capture the most recent and scientifically relevant evidence reflecting current knowledge and experimental approaches.

### 4.2 Study selection and search strategy

All research articles reporting how lncRNAs modulate glial cell expression in the context of NDs were selected. On the contrary reviews, systematic reviews, comments, and letters were excluded.

**Figure 2:**
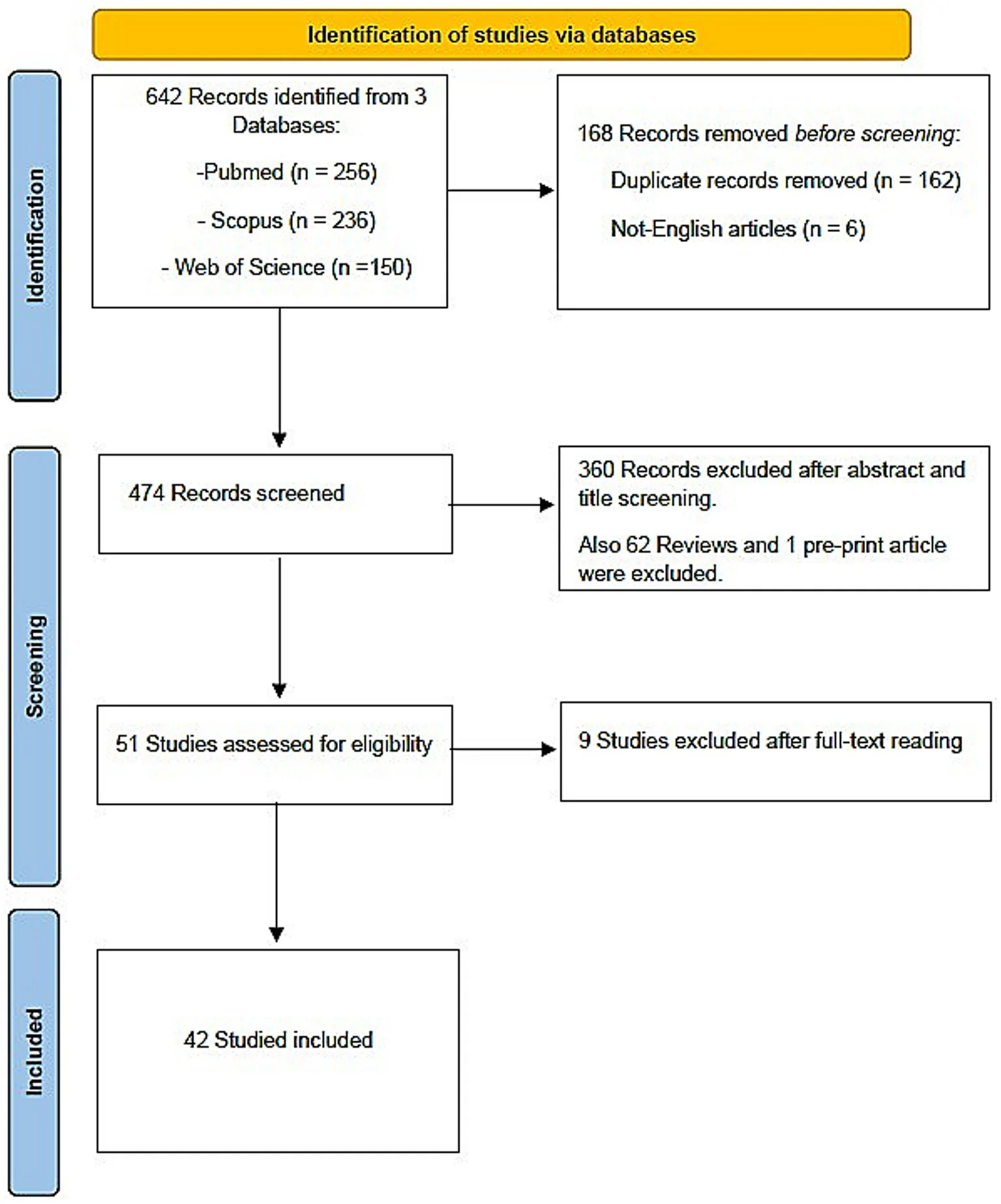
PRISMA flow diagram describing the study selection process.

Our focus was on the neuroinflammatory mechanisms of major NDs that impact cognition, such as AD, frontotemporal dementia, aging and PD. Diseases such as multiple sclerosis and stroke were excluded because, although they affect cognition, their etiology and pathophysiology are different. At first, search results were summarized, and duplicate citations were deleted, together with non-English articles. The titles were then examined to verify their relevance to the research target. Finally, the remaining articles’ abstracts were read, and those that did not meet the above eligibility criteria were excluded. The full text of all potentially relevant articles was then evaluated in depth. Figure 2 shows our study selection process. A total of 642 records were identified: 256 articles from PubMed database, 150 articles from Web of Science database and 236 articles from Scopus database. After removing 162 duplicates, 62 reviews, 6 non-English and 1 pre-print articles, 411 studies were identified. Later, 360 records were excluded by reading titles and abstracts, mainly because they were not focused on NDs. The 51 remaining studies were full-text screened.

Nine research articles were excluded from the discussion because they did not meet the inclusion criteria for the review. Thus, 42 articles have been included in the present study and reported in Tables 1–3. These ones summarize, for each study analyzed and grouped by disease, the main lncRNAs investigated by the various research groups on different experimental models, and the obtained results.

**Table 1:** Summary of studies on lncRNAs expression and their involvement in Alzheimer’s Disease pathways.

lncRNA	Glial cell type	Objective	Experimental model	Results	References
EPB41L4A-AS1	Astrocyte	Investigating the role of lncRNA EPB41L4A-AS1 in maintaining basal autophagy and modulating Aβ clearance by astrocytes	Human astrocytic U251	lncRNA EPB41L4A-AS1 expression is downregulated in AD patients, which inhibited transcription of multiple autophagy-related genes	Wang et al. (2024)
EPB41L4A-AS1	Astrocyte	Clarifying the role of this lncRNA on brain metabolism in relation to aging and cognitive decline	U251, SH-SY5Y cell line and Alzheimer dataset	Downregulation of EPB41L4A-AS1 reduces the expression of components of Complexes I-V of the respiratory chain, leading to a decrease in NAD + and ATP levels and thus to functional decline	Zhang et al. (2021)
MAGI2-AS3 and BACE1	Microglia	Studying the effects of certain lncRNAs on neuronal viability and neuroinflammation in *in vitro* models of AD. Evaluating their expression in serum samples from AD patients in relation to disease severity	SH-SY5Y, BV2, HEK293 cell line and serum samples from AD patients and healthy volunteers	Reduction of MAGI2-AS3 improves neuronal viability and attenuates neuroinflammation	Zhang and Wang (2021)
MALAT1	Microglia	Improvement of the inflammatory microenvironment, regulation of the microglial phenotype, and reduction of Aβ production	Mouse model, BEnd.3 and PC-12 cell line	Treatment with RVG-DDQ/PDP@siBACE1 nanoparticles improved cognitive performance in AD mice, reducing Aβ deposition and reducing neuroinflammation	Ke et al. (2024)
MEG3	Astrocyte	Clarifying the role and mechanisms of lncRNA MEG3 in rats with AD	Mouse model	Up-regulation of MEG3 inhibited astroglial activation, improved cognitive impairment, and alleviated neuronal damage	Yi et al. (2019)
MIAT	Microglia	Exploring the role of MIAT in LPS-induced microglial damage	BV2 cell line	MIAT/miR-613/NFAT5 axis promotes inflammation, whereas MIAT knockdown reduces apoptosis and the secretion of pro-inflammatory cytokines	Li et al. (2022)
NEAT1, KCNQ1OT1 and OIP5-AS1	Microglia	New insights into the pathogenesis of AD by studying the long RNA profiles of EVs	Human brain tissues *p.m.* from AD and healthy patients and BV2 cell line	AD-derived EVs significantly impaired Aβ phagocytosis in BV2 cells and primary microglia	Luo et al. (2023)
NEAT1 and MIAT	NA	Building lncRNA-associated ceRNA networks and revealing potential biomarkers in AD	In silico analysis	Identification of potential biomarkers and pathways in AD	Ou et al. (2021)
MIAT	Microglia	Exploring the role of MIAT in LPS-induced microglial damage	BV2 cell line	MIAT/miR-613/NFAT5 axis promotes inflammation, whereas MIAT knockdown reduces apoptosis and the secretion of pro-inflammatory cytokines	Li et al. (2022)
NONMMUT034127.2 and NONMMUT079254.1	Microglia and Astrocyte	Investigating lncRNA expression profiles in a mouse model of AD induced by chronic intranasal LPS	Mouse model induced by chronic intranasal LPS	Identification of 395 dysregulated genes linked to inflammation and apoptosis	Tang et al. (2019)
PRDM16-DT	Astrocyte	Determining if PRDM16-DT lncRNA regulates genes involved in synaptic function, apoptosis and inflammatory processes	Human brain tissues p.m. from AD and healthy patients, primary mouse astrocyte cultures and h uman iPSC-derived astrocytes	PRDM16-DT could be a potential drug target to ameliorate neurodegenerative processes associated with AD pathogenesis	Schröder et al. (2024)
PROX1-AS1 and AC073529.1	Not Available	Identifying lncRNAs differentially expressed between AD cases and controls and evaluating their potential as diagnostic markers	Blood samples from AD patients and healthy volunteers and in silico analysis	Expression of 28,909 lncRNAs was quantified. Identified 18 differentially expressed lncRNAs located in key AD-related genes	Mosquera-Heredia et al. (2024)
PRR34-AS1	Microglia	Determining the PRR34-AS1/miR-29c-3p axis in pathogenesis of AD	Serum samples from AD patients, BV2 and HMC3 cell line	PRR34-AS1 acts as a ceRNA, binding miR-29c-3p to regulate inflammation and microglial apoptosis	Cheng et al. (2025)
RN7SL1	Microglia	Evaluating the impact of RN7LS1 on the neuroimmune response	Human brain tissues p.m. from AD patients and BV2 cell line	RN7LS1 is upregulated in AD and correlates with disease severity, whereas its silencing reduces neuroinflammation	Liu et al. (2024)
SNHG14	Astrocyte	Investigating the potential molecular mechanisms by which Angiotensin-(1–7) modulates neuroinflammation in AD	APP/PS1 male transgenic mice and primary mouse astrocytes	Ang-(1–7) analog, AVE0991, inhibits astrocyte-mediated neuroinflammation through the SNHG14/miR-223-3p/NLRP3 pathway and provides neuroprotection in APP/PS1 mice	Duan et al. (2021)
SNHG14, MRTFA and MRTFB	Astrocyte and microglia	Studying the interaction between neurons and glial cells in Alzheimer’s progression through RNA sequencing data	In silico analysis	lncRNA-SNHG14, MRTFA and MRTFB co-expressing subclusters could create a pathogenic microenvironment in AD	Xie et al. (2024)
SOX2-OT	Microglia	Evaluating of the miR-143-3p/SOX2-OT axis in beta-amyloid-induced damage	SH-SY5Y, HMC3, cell line and serum samples from AD patients	SOX2-OT levels are elevated in the serum of AD patients; its silencing reduces neuroinflammation, apoptosis, and oxidative stress by sequestering miR-143-3p	Huang et al. (2025)
13 lncRNAs (e.g., GAS5, HOTAIR, LINC00152, MALAT1)	NA	Development of diagnostic and therapeutic tools focusing on neuroinflammation in AD	In silico analysis	Identification of signaling pathway with ability to modulate neuroinflammatory processes in AD	El Idrissi et al. (2021)

Aβ, amyloid-beta; AD, Alzheimer’s disease; Ang-(1–7), angiotensin-(1–7); APP/PS1, amyloid precursor protein/presenilin 1 transgenic mouse; BACE1, beta-site APP-cleaving enzyme 1; ceRNA, competing endogenous RNA; EVs, extracellular vesicles; HIF-1α, hypoxia-inducible factor 1-alpha; HPAs, human primary astrocytes; IL-1β, interleukin-1 beta; IL-6, interleukin-6; lncRNA, long non-coding RNA; LPS, lipopolysaccharide; miR/miRNA, microRNA; NA, not available; NFAT5, nuclear factor of activated T-cells 5; NLRP3, nod-like receptor protein 3; p.m., post-mortem; TNF-α, tumor necrosis factor-alpha.

**Table 2:** Summary of studies on lncRNAs expression and their involvement in Parkinson’s Disease pathways.

lncRNA	Glial cell type	Objective	Experimental model	Results	References
AK039862	Microglia	evaluating the role of lncRNAs in paraquat-induced neuroinflammatory damage	Mouse model, MN9D and BV2 cell line	Paraquat upregulates AK039862 which in turn acts as a driver in the neurodegeneration of dopaminergic neurons	Zhang et al. (2021)
DLX6-AS1	Microglia	Investigating the mechanism of DLX6-AS1 lncRNA on inflammatory responses in PD and evaluate its effect on neurological function and microglial inflammatory response	BV2 cell line and mouse model	DLX6-AS1 lncRNA promotes microglial inflammatory responses via the ceRNA network consisting of miR-223-3p and NRP1	Liu et al. (2022)
GAS5	Microglia	How GAS5 promotes the microglial inflammatory response by regulating the NLRP3 pathway through its “sponge” action on miR-223-3p	Mouse model	Down-regulation of GAS5 improved behavioral impairments in PD mice	Xu et al. (2020)
HOXA-AS2	Microglia	Analysis of lncRNA expression in PBMCs of PD patients to identify those involved in microglial polarization and its mechanism	Peripheral blood mononuclear cells (PBMCs) from PD patients and healthy volunteers, BV2 cell line and mouse model	HOXA-AS2 plays an important role in microglial polarization	Yang et al. (2021)
HOXA11-AS	Microglia	Investigating how HOXA11-AS and miR-124-3p modulate neuroinflammation in the PD model	SH-SY5Y, BV2 cell line and mouse model	Silencing of HOXA11-AS showed reduced microglial activation	Cao H. et al. (2021)
KCNQ1OT1	Microglia	Studying the function and potential mechanism of lncRNA KCNQ1 opposite strand/antisense transcript 1 (KCNQ1OT1) in the regulation of inflammasome activation in PD	BV2 cell line and mouse model	Silencing of KCNQ1OT1 suppressed activation of the microglial NLRP3 inflammasome and attenuated the loss of DAergic neurons improving motor function in PD mice	Li et al. (2024)
MALAT1	Microglia	Studying potential mechanisms leading to DAergic neuronal apoptosis and whether MALAT1 acts as a “sponge” for miR-23b-3p to regulate α-synuclein expression, influencing microglial autophagy and inflammatory response	MN9D and BV2 cell line	MALAT1 promotes α-synuclein expression by regulating miR-23b-3p, inducing microglial autophagy disorder and inflammation, leading to apoptosis of neurons	Geng et al. (2023)
MALAT1	Microglia	How MALAT1 influences inflammasome activation in PD via epigenetic suppression of Nrf2	BV2 cell line and mouse model	MALAT1 epigenetically inhibits NRF2, thereby inducing inflammasome activation and reactive oxygen species (ROS) production in PD mice	Cai et al. (2020)
MEG8	Microglia	How MEG8 may be implicated in PD progression by regulating neuronal inflammation	Blood sample from PD patients and healthy controls and BV2 cell line	MEG8 is notably reduced in PD and is a potential diagnostic biomarker for it	Lin et al. (2024)
MIR17HG	Microglia	Investigating the impact of MIR17HG lncRNA on viability, apoptosis, inflammatory factors, and oxidative stresses in mice and cells models	Blood samples from PD patients and healthy controls and mouse model. SH-SY5Y and BV2 cell line	Attenuation of MIR17HG reduced neuronal apoptosis and counteracted inflammatory response and oxidative stress in microglia	Zhang et al. (2022)
OIP5-AS1	Microglia	Investigating protective effect of OIP5-AS1	SH-SY5Y cell line	This lncRNA binds miR-137. This axis protects neurons by promoting mitochondrial autophagy and reducing α-synuclein accumulation	Zhao et al. (2022)
RMST	Astrocyte and microglia	Role of lncRNA RMST in DAergic neuronal damage in rats with PD	Rat model	RMST silencing reduced neuroinflammation and glial cell activation	Ma et al. (2021)
SINEUP	Astrocyte	Testing whether SINEUP non-coding RNA toward GDNF protects against motor deficits and neurodegeneration in mouse model of PD	Primary astrocyte cultures from mouse model	Development and validation of a synthetic miniSINEUP-GDNF, designed to increase GDNF protein levels as a therapeutic strategy for PD	Espinoza et al. (2020)
SNHG1	Microglia	Investigating the role and mechanism of SNHG1 in PD neuroinflammation	BV2 cell line, primary cortical neurons and mouse model	SNHG1 facilitates neuroinflammation in the progression of PD	Cao et al. (2018)
SNHG14	NA	How SNHG14 influences α-syn expression	Mouse model and MN9D cell line	lncRNA SNHG14 attenuates DAergic neuronal damage through downregulation of α-syn via miR-133b	Zhang et al. (2019)
TUG1	Microglia	Investigating the clinical value of lncRNA TUG1 in PD and its effect on the microglial inflammatory response	Blood sample from PD and AD patients and healthy controls, BV2 cell line and mouse model	TUG1 is dysregulated in PD patients and plays an important role in the early diagnosis of disease	Cheng et al. (2021)

α-Syn, alpha-synuclein; AD, Alzheimer’s disease; ceRNA, competing endogenous RNA; DAergic, dopaminergic; GDNF, glial cell line-derived neurotrophic factor; lncRNA, long non-coding RNA; miR/miRNA, microRNA; NA, not available; NLRP3, nod-like receptor protein 3; Nrf2, nuclear factor erythroid 2-related factor; NRP1, neuropilin-1; PBMCs, peripheral blood mononuclear cells; PD, Parkinson’s disease; ROS, reactive oxygen species.

**Table 3:** Summary of studies on lncRNAs expression and their involvement in aging – NDs pathways.

lncRNA	Glial cell type	Objective	Experimental model	Results	References
AK148321	Microglia	Investigating the function and regulation of lncRNA AK148321 in neuroinflammation	BV2 cell line	Overexpression of AK148321 inhibits LPS-induced microglial activation and expression of inflammatory cytokines	Gao et al. (2021)
KCNQ1OT1	Microglia	Discussing the function and potential mechanism of lncRNA KCNQ1OT1 in neuroinflammation	HMC3 cell line	Silencing of KCNQ1OT1 inhibited neuroinflammation	Song et al. (2021)
LMCD1-AS1 and ADAMTS9-AS1	Astrocyte	Investigating the therapeutic effect of astrocyte-derived EVs to mitigate neurotoxicity-induced transcriptomic changes	Human astrocytes and human brain endothelial cell culture	EVs transfer nucleic acids and proteins that change the lncRNA profile of recipient cells	Stewart et al. (2024)
LncRNA 4,344	Microglia	How lncRNA 4,344 modulates NLRP3-related neuroinflammation in cognitive decline	Rat model and rat microglia (RM) cell line	lncRNA 4,344 regulates NLRP3-related neuroinflammation and cognitive impairment by targeting miR-138-5p	Feng et al. (2021)
MEG3, MIR22HG, AC092687.3 and SDCBP2-AS1	Astrocyte	Identifying lncRNA/miRNA/mRNA axes that might be relevant to NDs progression	NHA cells and in silico analisys	22 potentially key axes and the relevance of 5 axes	Gil-Jaramillo et al. (2023)
MIR155HG, MIR210HG, Mir22hg, F630028O10Rik and MIR3142HG	Microglia	Activation and functioning of microglia according to different lncRNA expression profiles	iPSC-derived microglial cells and BV2 cell line	Increase in expression of inflammatory genes (Il1b, Ccl1 and Ccl4) in LPS-treated microglial cells	Kim et al. (2023)
NEAT1	Microglia	Role of NEAT1 in NLRP3 inflammasome activation in microglial cells	Mouse model C57BL/6 WT e KO for NEAT1 and murine N9 microglia	NEAT1 interacts with RNA-binding proteins to regulate NLRP3 inflammasome activation	Tastan et al. (2025)
Ptgs2os2, Bc1 e Morrbid	Microglia	Understand the processes involved in the regulation of microglial activity	Primary microglia from mouse	The combination of bzATP and LPS led to a sustained and robust inflammatory response	Zohar et al. (2023)
3222401L13Rik	Astrocyte	Identifying deregulated lncRNAs in glia during aging and determining whether their function is conserved in human astrocytes	Primary astrocyte cultures from mice and human induced pluripotent stem cell (iPSC)-derived astrocytes	The upregulation of 3222401L13Rik suggests that it acts as an adaptive mechanism in astrocytes to support neuronal function during aging	Schröder et al. (2025)

EVs, extracellular vesicles; iPSC, induced pluripotent stem cells; KO, knockout; lncRNA, long non-coding RNA; LPS, lipopolysaccharide; miR/miRNA, microRNA; mRNA, messenger RNA; NDs, neurodegenerative diseases; NHA, normal human astrocytes; NLRP3, nod-like receptor protein 3; RM, rat microglia; WT, wild-type.

## 5 LncRNA-mediated pathways in neurodegeneration

A balance between neuroprotective and pro-inflammatory functions of glial cells is critical in the progression of NDs (Liddelow et al., 2017). Notably, lncRNAs are complex and multifunctional modulators of glial responses in the brain. They influence neuroinflammation, astrocyte and microglial dysfunction, the clearance of toxic protein aggregates associated with NDs, particularly PD and AD, and synaptic functions (Yang et al., 2023). Recent literature on cognitive disorders mainly focuses on lncRNAs expressed or deregulated in astrocytes and microglia. Therefore, we focused on identifying lncRNAs involved in pathway dysregulated under conditions of astrocytosis or reactive microgliosis. The expression profiles of lncRNAs have been quantified, in the different studies, mainly using Next Generation Sequencing or microarray methods. Then, advanced bioinformatics and Machine Learning techniques were applied to identify those differently expressed in patients affected by NDs, compared to healthy patients (Mosquera-Heredia et al., 2024). Based on the literature review, we have categorized the lncRNAs in relation to the specific molecular signaling pathway affected.

### 5.1 Pathway of TLRs

TLRs are a group of pattern recognition receptors (PRRs) that are involved in the innate immune response. TLRs have a series of downstream effectors that ultimately lead to various cellular responses, including the production of proinflammatory cytokines and interferon (Heidari et al., 2022). TLRs are abundantly expressed by glial cells, and their activation can generate sustained immune responses (Lehnardt, 2010). The neuroprotective or neurotoxic effects characteristic of TLR activation depend on both the type and stage of NDs and the type of TLRs involved (Squillace and Salvemini, 2022). The signaling pathway activated by TLR7, TLR8, and TLR9, for example, appears to improve beta-amyloid uptake in microglia in the early stages of AD, but subsequently contributes to the process of neuroinflammation (Gambuzza et al., 2014). The activation of TLR4 by Aβ also activates microglia, triggering an increase in cytokines and oxidative stress (Hong et al., 2016), while the downregulation of TLR4 alleviates inflammation (Xu et al., 2020). Among the lncRNAs that act on TLR-mediated pathways, RMST is notable. Silencing RMST reduces the expression of TLRs 2 and 4 in neurons of a PD rat model. RMST actually promotes activation of the TLR/NF-κB pathway, evidenced by increased TLR2 and TLR4 levels in neurons and NF-κB nuclear translocation. Conversely, RMST suppression interrupts this cascade by reducing TLR2 and TLR4 expression and deactivating the NF-κB pathway. This results in reduced neuroinflammation, oxidative stress, and neuronal apoptosis. These changes translate into observable functional improvements in the animal model, suggesting that inhibiting RMST may represent a new therapeutic direction for PD (Ma et al., 2021).

### 5.2 Signaling of NF-κB

The transcription factor NF-κB is a key regulator of cellular differentiation, apoptosis, stress response, immune response and in the regulation of some pro-inflammatory cytokines, such as TNF*α* and IL-1β (Ke et al., 2024). Several lncRNAs are involved in the modulation of the NF-κB signaling pathway. As mentioned previously, RMST is involved in the damage to dopaminergic (DAergic) neurons, through the NF-κB signaling pathway. Indeed, RMST expression is increased in the brain tissue of PD rats, while its suppression reduces oxidative stress and neuronal apoptosis and improves neurobehavioral performance (Ma et al., 2021). In particular, RMST silencing was found to be associated with inhibition of the TLR/NF-κB signaling pathway, which could be related to the increase in the number of DAergic neurons, and thus the improvement in motor functions (Cheng and Zhu, 2019). Zhang J et al. investigated the impact of lncRNA MIR17HG in a mouse model of PD on the pro-inflammatory NF-κB/ inducible Nitric Oxide Synthase (iNOS)/ Cyclooxygenase-2 (COX2) pathways. They demonstrated that MIR17HG suppression mitigates microglia activation and protects DAergic neurons from the damage induced by the neurotoxin 6-OHDA, as a PD model. MIR17HG silencing hinders NF-κB phosphorylation and reduces COX2 and iNOS levels (Zhang et al., 2022). Additionally, the lncRNA HOTAIR has been implicated in epigenetic microglial activation. Under inflammatory conditions, HOTAIR expression is significantly upregulated and facilitates the activation of the NF-κB pathway, thereby promoting the transcription and release of key pro-inflammatory cytokines, such as TNF-α and IL-6 (El Idrissi et al., 2021; Obaid et al., 2018).

Furthermore, an innovative experimental model of AD—involving chronic intranasal administration of LPS—has demonstrated the induction of an immune response localized exclusively in the CNS, with high levels of cytokines in the cerebrospinal fluid but not in peripheral blood. As a result, 395 differentially expressed lncRNAs (172 upregulated and 223 downregulated) were identified in the hippocampus of the treated mice (Tang et al., 2019). NONMMUT034127.2 appears to be the most upregulated lncRNA, while NONMMUT079254.1 is the most downregulated, suggesting that they may be potential targets for understanding the pathogenesis of AD mediated by chronic inflammation (Tang et al., 2019).

### 5.3 Pathway of NLRP3 inflammasome

Inflammasomes are multiprotein complexes that mediate the inflammatory response triggered by a pathogen or induced by endogenous damage (Zhan et al., 2022). NLRP3 is the best characterized inflammasome; its activation leads to the release of numerous pro-inflammatory cytokines and to cell death by pyroptosis, a specific process of programmed cell death (Ramachandran et al., 2024). NLRP3 inflammasome-mediated neuronal pyroptosis is closely associated with the development of NDs, such as AD and PD. Aberrant activation of microglia and astrocytes contributes to the neuroinflammation typical of NDs, and NLRP3 is a key player in this process. Some lncRNAs modulate NLRP3 expression through ceRNA mechanisms (Han et al., 2023). One of the known negative regulators of NLRP3 expression is miR-223-3p (Mancuso et al., 2019).

Another lncRNA that can be considered a promoter of neuroinflammation at the level of microglia and cognitive impairment in rat models of LPS-induced damage is LncRNA 4,344, which, acting as a sponge for miR-138-5p, causes overexpression of NLRP3. The expression of lncRNA 4,344 is increased in the hippocampal tissues of rats and in microglia cells treated with LPS. Overexpression of lncRNA 4,344 enhances NLRP3 expression further, thereby exacerbating the inflammatory response and neuronal apoptosis (Feng et al., 2021). In contrast, silencing lncRNA 4,344 attenuates inflammatory lesions and alleviates cognitive impairment. For this reason, the researchers hypothesized that silencing this lncRNA could be a potential therapeutic approach for treating NDs. Also, GAS5 (Growth arrest-specific 5) promotes microglial inflammatory response by positively regulating the NLRP3 pathway through “sponging” miR-223-3p. In fact, in a mouse model of PD it has been shown that GAS5 is upregulated in activated microglia, while its silencing suppresses disease progression (Xu et al., 2020).

Another lncRNA involved in inflammasome regulation is SNHG1 (Small Nucleolar RNA Host Gene 1) which functions as a ceRNA for miR-7, increasing the expression of NLRP3. This axis was studied by Cao B. and colleagues (Cao et al., 2018) who highlighted how the reduced expression of miR-7 caused by the action of SNHG1 led to the activation of the NLRP3 inflammasome. On the other hand, silencing SNHG1 results in an increase in miR-7 levels, the suppression of microglial activation and NLRP3 levels, and thus a reduced loss of DAergic neurons, in MPTP-induced mouse model of PD.

LncRNA KCNQ1OT1 promotes the activation of the NLRP3 inflammasome and causes the loss of DAergic neurons in the mouse model with MPTP/Probenecid-Induced PD. This lncRNA is localized in both the cytosol and nucleus of microglia. KCNQ1OT1 is a dual regulator of NLRP3, acting both in the cytosol, as a ceRNA for miR-186, and in the nucleus. MiR-186 binds to the messenger RNA of NLRP3, decreasing its expression. KCNQ1OT1 sponges miR-186, thereby promoting inflammasome activation. In the nucleus, KCNQ1OT1 interacts with the RNA-binding protein DGCR8 (DiGeorge syndrome Critical Region gene 8), which is involved in the maturation of pri-miR-186. With this upstream mechanism, KCNQ1OT1 is able to maintain high levels of NLRP3. These two modes of regulation by KCNQ1OT1 converge to promote NLRP3 inflammasome activation in microglia, contributing to the loss of DAergic neurons in PD. Conversely, it has been shown that downregulation of KCNQ1OT1 suppresses NLRP3 activation in microglia, thereby attenuating DAergic neuronal loss and improving motor function (Li et al., 2024). Another *in vitro* experiment on microglial cells showed that inhibition of KCNQ1OT1 reduces neuroinflammation and neuronal apoptosis through regulation of the axis miR-30e-3p/NLRP3 (Song et al., 2021).

Similarly, HOXA11-AS promotes neural damage via the production of pro-inflammatory interleukins, in a mouse model of PD. This is related to the activation of microglia through the miR-124-3p-FSTL1-NF-κB axis. MiR-124-3p plays an important role in mitigating neuronal damage and microglia activation (Cao H. et al., 2021). This miRNA suppressed FSTL1, a protein involved in numerous pathways, including the inflammation, in association with NF-κB (Hu et al., 2019). Although the role of FSTL1 is controversial, it has been shown that overexpression of HOXA11-AS supported the expression of FSTL1. Furthermore, inhibition of HOXA11-AS significantly increased the levels of miR-124-3p, attenuating the axis FSTL1/NF-κB, and consequently NLRP3 expression. Overall, this results in neuroprotective effects, thereby mitigating disease progression (Cao H. et al., 2021). DLX6-AS1 promotes microglial inflammatory response in a mouse model of PD, through the ceRNA network consisting of miR-223-3p and Neuropilin-1 (NRP1) (Liu et al., 2022). The role of miR-223-3p is to safeguard neurons against degeneration, to minimize NLRP3-induced inflammation and to restrict pyroptosis (Cihankaya et al., 2024). Therefore, its overexpression mediates neuroprotection (Morquette et al., 2019). NRP1 is a multifunctional protein involved in axonal development (Graziani and Lacal, 2015), whose expression is significantly increased in patients with cognitive decline (Bosseboeuf and Raimondi, 2020). A study by L. Liu showed that miR-223-3p can directly bind to NRP1 mRNA, thereby decreasing its levels. DLX6-AS1 influences NRP1 levels through a ceRNA mechanism involving miR-223-3p. This lncRNA reduces the amount of available miR-223-3p, while increasing the levels of NRP1, thereby promoting inflammation. Furthermore, in LPS-induced microglial cells, the reduction of miR-223-3p levels has been found to induce an increase in NLRP3 inflammasome expression. On the other hand, silencing DLX6-AS1 leads to a decrease in NLRP3 inflammasome levels (Liu et al., 2022). Overall, the overexpression of DLX6-AS1 induces a reduction in miR-223-3p, which leads to an increase in both NRP1 levels and in NLRP3 activity, contributing to neuroinflammation (Liu et al., 2022). To study pathways that modulate the glia inflammatory phenotype in vitro, the cells are typically treated with lipopolysaccharide (LPS). Thus, it has been found that the overexpression of AK148321 (murine lncRNA) inhibits the activation and production of inflammatory cytokines in activated microglia cells (BV2) (33301806). AK148321 acts as a ceRNA by inhibiting miR-1199-5p, which in turn regulates the expression of HSPA5, a heat shock protein that modulates the folding and stabilizes NLRP3 (von Herrmann et al., 2018). Downregulation of HSPA5 leads to increased expression of NLRP3 in LPS-stimulated microglial cells. The reciprocal regulation between NLRP3 and HSPA5 is influenced upstream by the axis AK148321/miR-1199-5p. In fact, the reduction of AK148321, induced by LPS, releases miR-1199-5p, which in turn suppresses the expression of HSPA5. Instead, the overexpression of AK148321 sequesters miR-1199-5p, attenuating the inhibition on HSPA5 and leading to an increase in HSPA5 levels (Gao et al., 2021). The neuroprotective function of the AK148321/miR-1199-5p/HSPA5 regulatory network was investigated on a BV2 microglia/HT22 hippocampal neuron co-culture system. The stimulation with LPS on BV2 cells—transfected with AK148321—inhibits apoptosis in HT22 cells, whereas it promotes HT22 cell apoptosis when HSPA5 is inhibited. Thus, in a murine model, it has been demonstrated that the axis AK148321/miR-1199-5p/HSPA5 regulates the activation of microglial cells, the production of inflammatory cytokines and modulates the apoptosis of neuronal cells (Gao et al., 2021). It is not certain whether the human homolog of AK148321, ANRIL, acts similarly. Furthermore, the role of ANRIL appears to vary depending on the pathological and cellular context. In various inflammatory situations, ANRIL overexpression promotes inflammation, while its inhibition alleviates inflammation and neuronal damage (Ge et al., 2019; Feng et al., 2019).

On the other hand, MEG3 is a lncRNAs associated with inflammation (Na et al., 2020). The role of MEG3 has also been explored in a rat model of AD, to determine its correlation with inflammatory damage and astrocytic activation. The up-regulation of MEG3 suppressed oxidative stress, astrocyte activation, decreased the expression of Aβ in the rat hippocampus and the expression of pro-inflammatory cytokines. MEG3 acts by inactivating the PI3K/Akt signaling pathway, whose expression of phosphorylated forms decreases significantly. This not only helps alleviate neuronal damage, but also improves cognitive decline (Yi et al., 2019). The role of MEG8 in NDs has also been explored. It was found that PD patients have higher levels of MEG8 compared with healthy individuals, suggesting that this lncRNA plays a role in the progression of the disease and could be a potential diagnostic biomarker. Experiments on microglia activated with LPS showed that the overproduction of pro-inflammatory factors decreased in the case of MEG8 overexpression, but it is suppressed by the action of miR-485-3p. Therefore, it is assumed that MEG8 acts as a sponge for miR-485-3p, mitigating inflammatory damage in PD (Lin et al., 2024). Another lncRNA closely associated with the manifestation of PD is TUG1, which has been found in the serum of PD patients compared to healthy controls. *In vitro* studies on microglia cells and on mouse models have shown that TUG1 expression is significantly up-regulated in both PD mice and activated microglia cells, while down-regulation of TUG1 inhibits the release of pro-inflammatory factors such as IL-6, IL-1β, and TNF-*α* (Cheng et al., 2021). HOXA-AS2 promotes neuroinflammation as it regulates microglial activation through the recruitment of the Polycomb Repressive Complex 2 (PRC2) and histone modification of the Peroxisome-proliferator-activated receptor-Gamma Coactivator 1-alpha (PGC-1*α*) promoter. PGC-1α is the co-activator of numerous genes involved in energy metabolism. HOXA-AS2 levels were shown to be significantly increased in PD patients, while PGC-1α expression was repressed. Silencing HOXA-AS2, on the other hand, promotes a pro-reparative microglia status, by PGC-1α (Yang et al., 2021).

### 5.4 The TREM2-NLRP3-DAM regulatory axis and cell death crosstalk in Parkinson’s disease

To fully address the complexity of microglial heterogeneity and receptor-mediated signaling checkpoints in neurodegeneration, it is crucial to integrate the emerging role of the Triggering Receptor Expressed on Myeloid Cells 2 (TREM2) (Zhang et al., 2025; Huang et al., 2024; Liu et al., 2025). Recent landmark studies have elucidated a sophisticated molecular bridge linking TREM2 deficiency to the hyperactivation of the NLRP3 inflammasome and microglial phenotypic shifts in Parkinson’s disease (PD) models (Zhang et al., 2025; Huang et al., 2024; Liu et al., 2025).

*In vivo* experiments using a chronic MPTP-induced PD mouse model combined with stereotactic injection of AAV-TREM2-shRNA into the bilateral substantia nigra demonstrated that TREM2 knockdown profoundly aggravates the loss of tyrosine hydroxylase (TH)-positive dopaminergic neurons and accelerates motor dysfunction, as validated by the traction and rotarod tests (Huang et al., 2024). Mechanistically, dissection in LPS/ATP-stimulated BV2 microglial cells revealed that TREM2 deficiency triggers an upregulation of the upstream TLR4/MyD88 complex, which in turn drives the robust phosphorylation and nuclear translocation of NF-κB (Huang et al., 2024). This unregulated priming step leads to aberrant NLRP3 inflammasome assembly, marked by severe increases in cleaved caspase-1, elevated secretion of mature IL-1β and IL-18, and the execution of microglial pyroptosis via the cleavage of Gasdermin D into its active form (GSDMD-N) (Huang et al., 2024).

Concurrently, high-throughput transcriptomic and molecular analyses have redefined how TREM2 regulates the fine-grained sub-phenotypes of microglia, directly addressing the limitations of the obsolete, rigid M1/M2 framework (Zhang et al., 2025; Liu et al., 2025). In an A53T *α*-synuclein (α-Syn) transgenic mouse model of PD, hippocampal silencing of TREM2 via adeno-associated virus transfection was shown to cause pronounced synaptic loss, severe cognitive impairment in the novel object recognition and Morris water maze tests, notably driving neuronal and synaptic damage without exacerbating the *in vivo* aggregation of pathological α-synuclein (Zhang et al., 2025). At the single-cell level, TREM2 acts as a mandatory molecular switch governing Disease-Associated Microglia (DAM) dynamics (Zhang et al., 2025). Upon stimulation with α-Syn preformed fibrils (PFF), TREM2 deficiency dramatically suppresses the protective, anti-inflammatory DAM transcriptomic signature while actively accelerating a highly neurotoxic, pro-inflammatory DAM phenotype through the sustained co-activation of the ERK1/2 (MAPK) and NF-κB pathways (Zhang et al., 2025).

Importantly, this microglial-driven neuroinflammatory microenvironment does not operate in isolation but participates in a complex, self-amplifying network of interconnected programmed cell death modalities (Liu et al., 2025). As evidenced by recent redox biology mapping in PD, chronic neuroinflammation and mitochondrial failure serve as central hubs where NLRP3/GSDMD-mediated pyroptosis actively cross-talks with caspase-dependent apoptosis, RIPK1/RIPK3/MLKL-driven necroptosis, and lipid peroxidation-induced ferroptosis stemming from glutathione peroxidase 4 (GPX4) inactivation (Liu et al., 2025). Crucially, this interplay between autophagic clearance and ferroptosis is finely orchestrated by shared upstream signaling regulators, most notably the p53 and AMPK pathways. Under severe oxidative stress, AMPK activation functions as a metabolic rheostat that promotes autophagic flux to clear protein aggregates while concurrently modulating lipid peroxidation. Parallelly, p53 signaling acts as a critical molecular executioner that can transcriptionally downregulate cystine/glutamate antiporter components (such as SLC7A11), thereby directly bridging autophagic vacuole accumulation to ferroptotic cell death pathways in dysregulated glial environments.

The continuous release of pro-inflammatory cytokines (TNF-α, IL-1β) by dysregulated, non-homeostatic microglial states further sustains cellular redox imbalance, locking dopaminergic systems into a multi-pathway death loop (Liu et al., 2025). Consequently, understanding how emerging non-coding RNAs modulate upstream regulators like TREM2 or downstream effectors within the TLR4/NF-κB and ERK1/2 axes represents a crucial frontier for disruptive therapeutics.

### 5.5 Comprehensive profiles of multifunctional glial lncRNA hubs: NEAT1, MALAT1, and SNHG14

To provide a non-fragmented overview of the most heavily investigated non-coding transcripts in this field, this section consolidates the multi-targeted molecular actions of NEAT1, MALAT1, and SNHG14, which cross-talk simultaneously with inflammatory cascades, clearance pathways, and synaptic stability.

In the context of crosstalk between microglia and dopaminergic neurons, a paracrine mechanism involving the lncRNA AK039862 that induces cell death has been demonstrated (Zhang et al., 2021). In particular, mouse models and cells stimulated with paraquat—an environmental pollutant that induces PD—are capable of altering the expression of AK039862 and Pafah1b1 in neurons, thereby inducing cell death.

Paraquat upregulates AK039862, which acts as a driver of neuroinflammation and the degeneration of dopaminergic neurons by influencing the post-transcriptional Pafah1b1/Foxa1 pathway (Zhang et al., 2021).

#### 5.5.1 NEAT1 (nuclear enriched abundant transcript 1)

NEAT1 acts as a critical agent that intensifies the inflammatory response while tightly modulating cellular homeostasis and structural plasticity. In the context of neuroinflammation, NEAT1 directly promotes the cascade triggered by NLRP3 inflammasome activation in microglia. *In vitro* studies on murine N9 microglia cells and corresponding *in vivo* models demonstrate that NEAT1 interacts with specific RNA-binding proteins to orchestrate NLRP3 activation and contribute to microglial cell death; notably, its knockout in C57BL/6 mice or siRNA-mediated silencing significantly attenuates NLRP3-dependent signaling (Tastan et al., 2025). Parallelly, NEAT1 exercises extensive epigenetic control within the nucleus, where it modulates neuronal histone methylation, dynamically altering chromatin architecture and transcription factor accessibility (Butler et al., 2019). Through these epigenetic modifications near the transcription start site (TSS), NEAT1 induces autophagy by regulating the transcription of key endocytic and autophagic genes, including atg5, atg3, and beclin1, in neuroglial cells. While its expression is found significantly reduced during the early stages of Alzheimer’s disease (AD) in mouse models—potentially explaining early defects in autophagic Aβ clearance (Wang et al., 2022)—network bioinformatics analyses reveal that NEAT1 is concurrently relevant to synaptic function, showing tight associations with chronic astrocyte reactivity and memory deficits in advanced disease stages (Irwin et al., 2023; Li and Wang, 2023).

#### 5.5.2 MALAT1 (metastasis-associated lung adenocarcinoma transcript 1)

MALAT1 operates as a highly specialized master regulator across different cell lines and neurodegenerative models, directly linking immediate immune responses to sophisticated epigenetic silencing. In the NF-κB signaling pathway, *in vitro* experiments on dopaminergic neuronal cells and microglia demonstrate that MALAT1 overexpression induces the nuclear translocation of the NF-κB p65 subunit, driving a robust microglial pro-inflammatory response (Geng et al., 2023). This transcript is heavily upregulated in both mouse model brains and lipopolysaccharide (LPS)-stimulated microglial cells, whereas its silencing exerts a protective effect against microglia-mediated neuronal damage (Cai et al., 2020). Beyond direct transcription factor modulation, MALAT1 facilitates neuroinflammation through complex epigenetic mechanisms in MPTP-induced Parkinson’s disease (PD) models, including BV2 murine microglia and N2a human neuronal cells. Here, MALAT1 recruits the polycomb repressive complex 2 (PRC2) component EZH2 to the Nrf2 gene promoter, leading to an increased accumulation of the repressive H3K27me3 histone mark. This epigenetic silencing of Nrf2 suppresses downstream antioxidant defenses, exacerbating ROS production and triggering secondary NLRP3 inflammasome activation (Cai et al., 2020). In processing and cell survival, MALAT1 acts as a competing endogenous RNA (ceRNA) network hub. In PC-12 neuronal cells treated with Aβ1-42 (AD model), it sponges miR-181c, which normally suppresses pro-apoptotic targets, causing an upregulation of BCL2L11 and promoting neuronal apoptosis (Ke et al., 2024). In PD models, it functions via the miR-23b-3p/*α*-synuclein axis, where its “sponging” activity increases α-synuclein levels, inducing microglial autophagy dysfunction and dopaminergic cell death (Geng et al., 2023). Finally, MALAT1 directly influences cognitive networks by modulating the expression of multiple synaptic genes, with its downregulation being associated with reduced dendritic and synaptic density (Bernard et al., 2010).

#### 5.5.3 SNHG14 (small nucleolar RNA host gene 14)

SNHG14 represents a predominantly cytoplasmic lncRNA that coordinates pathological microenvironments and proteinopathy progression in both astrocytes and microglia. In astrocytes, where it is highly enriched in the cytoplasm, SNHG14 acts as a known negative regulator of neuroprotection by directly binding to miR-223-3p; this competitive sequestration reduces available miRNA levels, subsequently triggering an upregulation of the NLRP3 inflammasome and exacerbating the astrocytic inflammatory state (Duan et al., 2021). In AD models, single-nucleus RNA sequencing and molecular docking analyses have identified SNHG14 as part of a distinct SNHG14_MRTFA_MRTFB regulatory subcluster co-expressed by clusters of neurons and glial cells, which contributes to local microenvironment remodeling, leading to apoptosis dysregulation and accelerated neuronal degeneration (Xie et al., 2024). Furthermore, SNHG14 directly drives dopaminergic injury in PD pathology. In mouse models and MN9D cells treated with rotenone, SNHG14 competes with miR-133b to regulate *α*-synuclein expression. By functioning as a molecular sponge for miR-133b, SNHG14 prevents the miRNA from suppressing its target, leading to a profound increase in α-synuclein accumulation and accelerating dopaminergic neuronal death, while its targeted interference successfully mitigates this cascade (Zhang et al., 2019).

#### 5.5.4 MIAT (myocardial infarction associated transcript)

The lncRNA MIAT emerges as a central regulator involved in both the microglial inflammatory response and cerebral microcirculation (Li et al., 2022). In LPS-stimulated mouse cells, MIAT expression increases significantly, acting as a sponge for miR-613 and thereby releasing the transcription factor NFAT5 (Nuclear Factor of Activated T-cell 5). Hyperactivation of the MIAT/miR-613/NFAT5 axis promotes both the secretion of pro-inflammatory cytokines and microglial apoptosis (Li et al., 2022). Furthermore, MIAT is considered a promising biomarker for the diagnosis and longitudinal monitoring of AD (Gil-Jaramillo et al., 2023).

### 5.6 Context-dependent and stage-specific dynamics of glial lncRNAs

To fully understand the therapeutic potential of lncRNAs like MEG3 and ANRIL, it is essential to address their highly context-dependent and seemingly contradictory actions across different neuropathological microenvironments. For instance, while MEG3 expression exerts neuroprotective effects in Alzheimer’s disease models by suppressing localized neuroinflammation and amyloid cytotoxicity, its targeted inhibition has paradoxically been reported as protective against ischemic stroke injury. Similarly, ANRIL exhibits dual, opposing roles depending on the specific activation state of downstream inflammatory loops and cell types involved. These discrepancies underscore that glial lncRNAs do not function as rigid, binary “pro-” or “anti-inflammatory” switches. Instead, their biological outcomes are strictly contingent upon the prevailing cellular context, chromatin accessibility, and the specific stage of the neurodegenerative process, representing a pivotal consideration for any translation into targeted therapeutics.

Furthermore, the functional profile of glial lncRNAs exhibits strict temporal and stage-specific dynamics during neurodegeneration. Rather than maintaining a permanent pro-inflammatory or neurotoxic role, certain transcripts undergo a functional shift. In the early asymptomatic stages of proteinopathy, specific lncRNAs actively prime microglia and astrocytes toward beneficial activation states, promoting effective Aβ plaque clearance, protein aggregate phagocytosis, and synaptic support. However, as the disease transitions into advanced, chronic phases, the persistent upregulation of these identical non-coding networks triggers a maladaptive, self-amplifying neuroinflammatory loop that overcomes homeostatic defenses and drives severe synaptic stripping and neuronal death. Distinguishing these time-dependent dualities is mandatory to avoid detrimental off-target effects in drug development.

## 6 Autophagy and apoptosis

Apoptosis is a cell death process finely regulated. In fact, excessive and deregulated apoptosis can lead to various pathological conditions, including the onset of NDs (Elmore, 2007). Autophagy, on the other hand, is a catabolic process essential for maintaining cellular homeostasis. Through this mechanism, cells are able to degrade and recycle proteins and organelles (Liu et al., 2023). In the context of neuroinflammation and neurodegeneration, autophagy plays a significant role in the clearance of both Aβ (Kim et al., 2024) and *α*-synuclein (Choi et al., 2020). Some lncRNAs regulate the autophagy process, influencing the course of the disease. EPB41L4A-AS1 is a lncRNA implicated in several pathological processes. It has also been correlated with aging and is deregulated in patients with AD (Yang et al., 2021). In particular, down-regulation of EPB41L4A-AS1 was found to prevent Aβ clearance through a complex mechanism involving the transcription of autophagy-related genes and modulation of lysosomal activity. This lncRNA positively regulates the activity of GCN5L2 (General Control of Amino Acid Synthesis 5-like 2), a protein that functions as a histone acetyltransferase, responsible for several epigenetic modifications near the transcription start sites of target genes (Wang et al., 2024). EPB41L4A-AS1 is necessary to maintain the transcription of autophagy-related genes (ATG), which regulate autophagosome formation (Gianchecchi et al., 2014). Furthermore, it plays a role in maintaining lysosomal function, as silencing EPB41L4A-AS1 also inhibits the expression of lysosomal markers such as LAMP1, CTSB, and CTSD (Wang et al., 2024). Therefore, EPB41L4A-AS1 promotes Aβ clearance through the autophagic-lysosomal pathway. In contrast, its downregulation, as observed in AD patients, leads to failure in Aβ clearance. A study conducted by Yang T. et al. highlights that EPB41L4A-AS1 expression decreases with age and identifies this lncRNA as a metabolic regulator that acts epigenetically on the transcription of the NMNAT2 gene (an enzyme crucial for NAD + synthesis, predominantly expressed in the brain), causing a decrease in its expression. Therefore, aging induces a reduction in EPB41L4A-AS1, which in turn compromises the synthesis of NAD + and ATP. Since the brain has high energy requirements, this metabolic deficit accelerates physiological aging and actively promotes the onset and progression of NDs (Yang et al., 2021). An interesting lncRNA found at elevated levels in the serum of AD patients is SOX2-OT (Huang et al., 2025). It has been observed that in neurons and microglial cells, SOX2-OT promotes amyloid-induced apoptosis, while its silencing counteracts cellular damage by increasing the levels of the anti-apoptotic protein Bcl-2 and reducing the expression of pro-apoptotic factors (Huang et al., 2025).

Another modulator of PD is lncRNA miR-17-92a-1 cluster host gene (MIR17HG). This is significantly upregulated both in cellular and animal models of the disease, and also in the plasma of PD patients. MIR17HG induces neuronal apoptosis by acting on the miR-153-3p. This miRNA binds directly to the messenger of the SNCA gene that codes for α-synuclein. In fact, the overexpression of miR-153-3p leads to a reduction in α-synuclein levels. MIR17HG, on the other hand, binds miR-153-3p inhibiting its function, leading to an increase in α-synuclein with consequent neuronal apoptosis and reactive microgliosis (Zhang et al., 2022).

An *in vitro* study using SH-SY5Y neuroblastoma cells treated with MPP + as a PD model shows that Opa-interacting protein 5 antisense RNA 1 (OIP5-AS1) expression is significantly reduced in the pathological context. The role of OIP5-AS1 is crucial for the survival of DAergic neurons. OIP5-AS1 acts as a sponge for miR-137, leading to increased expression of the downstream target gene NIX, which promotes selective mitophagy of damaged mitochondria, reduces ROS levels, and prevents apoptosis (Zhao et al., 2022). In PD, the downregulation of OIP5-AS1 actively contributes to disease progression by depriving cells of an essential protective mechanism.

## 7 Synaptic plasticity

Synaptic plasticity is a fundamental and dynamic mechanism that allows neurons to change their connections and functionality in response to different stimuli (Mohammadi et al., 2022). Glial cells assume a pivotal function in the synaptic process and its modulation (Citri and Malenka, 2008). In particular, the synaptic plasticity processes can also be directly or indirectly influenced by glial lncRNA.

### 7.1 Astrocytic signaling pathways in synaptic stripping: JAK–STAT and the complement cascade

To provide a more balanced overview of glial contributions and address the specific molecular cascades driving astrocyte reactivity, it is essential to delineate the role of the JAK–STAT pathway and the complement system in synaptic plasticity. Reactive astrogliosis is fundamentally governed by the activation of the Janus kinase/signal transducer and activator of transcription (JAK–STAT) pathway, which acts as a primary transcriptional driver of structural and functional remodeling in astrocytes during neurodegeneration. Once triggered by microglial-derived pro-inflammatory factors, the JAK–STAT cascade induces a persistent reactive state, upregulating intermediate filaments and altering the astrocytic secretome. Concurrently, this reactive transformation triggers the aberrant activation of the classical complement cascade, specifically marked by the upregulation of complement component C3 and its receptor C3aR. In neurodegenerative microenvironments, astrocyte-derived C3 binds to neuronal complement receptors, tagging vulnerable synapses for microglial-mediated phagocytosis and elimination (synaptic stripping). Disrupting this astrocyte-specific JAK–STAT/C3-C3aR axis represents a critical therapeutic target to mitigate early synaptic loss and cognitive decline before irreversible neuronal apoptosis occurs.

For example, PRDM16-DT is a lncRNA expressed mainly by astrocytes, and its expression has been found to be significantly lower in post-mortem brain tissue from AD patients than in healthy individuals. It is involved in the regulation of genes that control neuronal support and synaptic organization. Its silencing, in fact, compromises the ability of astrocytes to support neuronal function, leading to a decrease in neuronal viability and dendritic spine density (Schröder et al., 2024).

Another lncRNA, 3222401L13Rik, whose human homolog is ENSG00000272070, was found upregulated in the astrocytes of aged mice. This lncRNA appears to play a role in regulating genes essential for neuronal support and synaptic organization. Its silencing compromises both glutamate uptake and Ca2 + ion signaling in astrocytes, resulting in damage to the plasticity of neuronal networks and a decrease in synaptic density (Schröder et al., 2025). Furthermore, it has been shown that 3222401L13Rik acts by modulating the transcription factor Neuronal PAS Domain Protein 3 (Npas3), which regulates the expression of numerous target genes related to neuronal development (Schröder et al., 2025; Luoma and Berry, 2018).

## 8 Fatty acid metabolism

Metabolic dysfunctions are well recognized as contributing to the pathogenesis of NDs (Asimakidou et al., 2025; Muddapu et al., 2020). In particular, the interaction between lncRNAs and lipid metabolism influences the homeostasis of glial cells (Chen et al., 2021). Metabolic dysfunction emerges as a key etiological factor in NDs. Excess peripheral lipids, particularly lipotoxicity induced by palmitic acid (PA), impair astrocyte function. This state of metabolic stress induces reprogramming of ceRNA networks, resulting in the activation of pathological pathways that lead to chronic neuroinflammation and neuronal death, ultimately promoting the pathogenesis of conditions such as AD and PD (Asimakidou et al., 2025; Muddapu et al., 2020). Some lncRNAs appear to be specifically involved in these pathways. Bioinformatic analysis showed that MEG3 is downregulated under lipotoxic conditions in astrocytes (Gil-Jaramillo et al., 2023). Some studies have linked MEG3 overexpression with improved cognitive impairment and anti-apoptotic action (Yi et al., 2019). In contrast, other studies have shown that inhibition of MEG3 protects against neuronal death following an ischemic event (Yan et al., 2017). This could be due to the numerous isoforms of MEG3, generated by alternative splicing, and with distinct regulatory roles depending on the context, tissue type, and nuclear/cytoplasmic localization (Yi et al., 2019; Yan et al., 2017). Therefore, MEG3 is an interesting biomarker and potential therapeutic target, especially in NDs (Gil-Jaramillo et al., 2023; Farhadieh and Ghaedi, 2023). Exposure to palmitic acid reprograms astrocytes by deregulating ceRNA networks. The overexpressed lncRNA AC092687.3 acts as a pro-degenerative activator, promoting AD by activating axes that dysregulate cholesterol metabolism and induce the programmed cell death of astrocytes. Meanwhile, SERTAD4-AS1, which controls miRNAs related to fatty acid biosynthesis, is downregulated, negatively affecting lipid metabolism. The role of the SDCBP2-AS1 lncRNA appears to be more complex: it may have a protective function against apoptosis, but it also activates a specific axis that promotes inflammation and cell death, thereby contributing to the pathogenesis of AD. In summary, this intricate network of lncRNAs in astrocytes mediates the transition from a protective state to a neurotoxic state in response to metabolic stress (Gil-Jaramillo et al., 2023). However, the astrocytic response in lipotoxic conditions still needs to be further investigated further.

## 9 Other biological outcomes

lncRNAs can act as post-transcriptional regulators of gene expression by interacting with miRNAs, both as sponges and enhancers, increasing the expression of target genes (Alessio et al., 2020). In the context of AD, the role of MAGI2-AS3 has been explored in relation to the enzyme beta-secretase 1 (BACE1), a transmembrane protein particularly expressed in neurons and astrocytes (Hampel et al., 2021). This enzyme is capable of cleaving the Amyloid Precursor Protein (APP) into A*β* fragments that form aggregates in AD (Hampel et al., 2021). miRNA-374b-5p acts directly on BACE1 by binding to the 3’ UTR of its mRNA. MAGI2-AS3, on the other hand, acts as a sponge for miRNA-374b-5p. Increased levels of MAGI2-AS3 therefore lead to increased expression of BACE1. This has been observed both *in vitro* studies and in the serum of AD patients. Furthermore, in vitro studies on neuronal cells and microglia have shown that MAGI2-AS3 knockdown increases neuronal viability and attenuates neuroinflammation (Yang et al., 2021). Another example of post-transcriptional regulation on the BACE1 enzyme is mediated by the lncRNA BACE1-AS. This lncRNA is transcribed as the antisense RNA from the same gene encoding BACE1. By binding to the enzyme’s mRNA, BACE1-AS increases its stability because it masks the binding site for miR-485-5p. This leads to an increased presence of Aβ aggregates and consequently greater neuronal damage (Faghihi et al., 2008).

**Figure 3:**
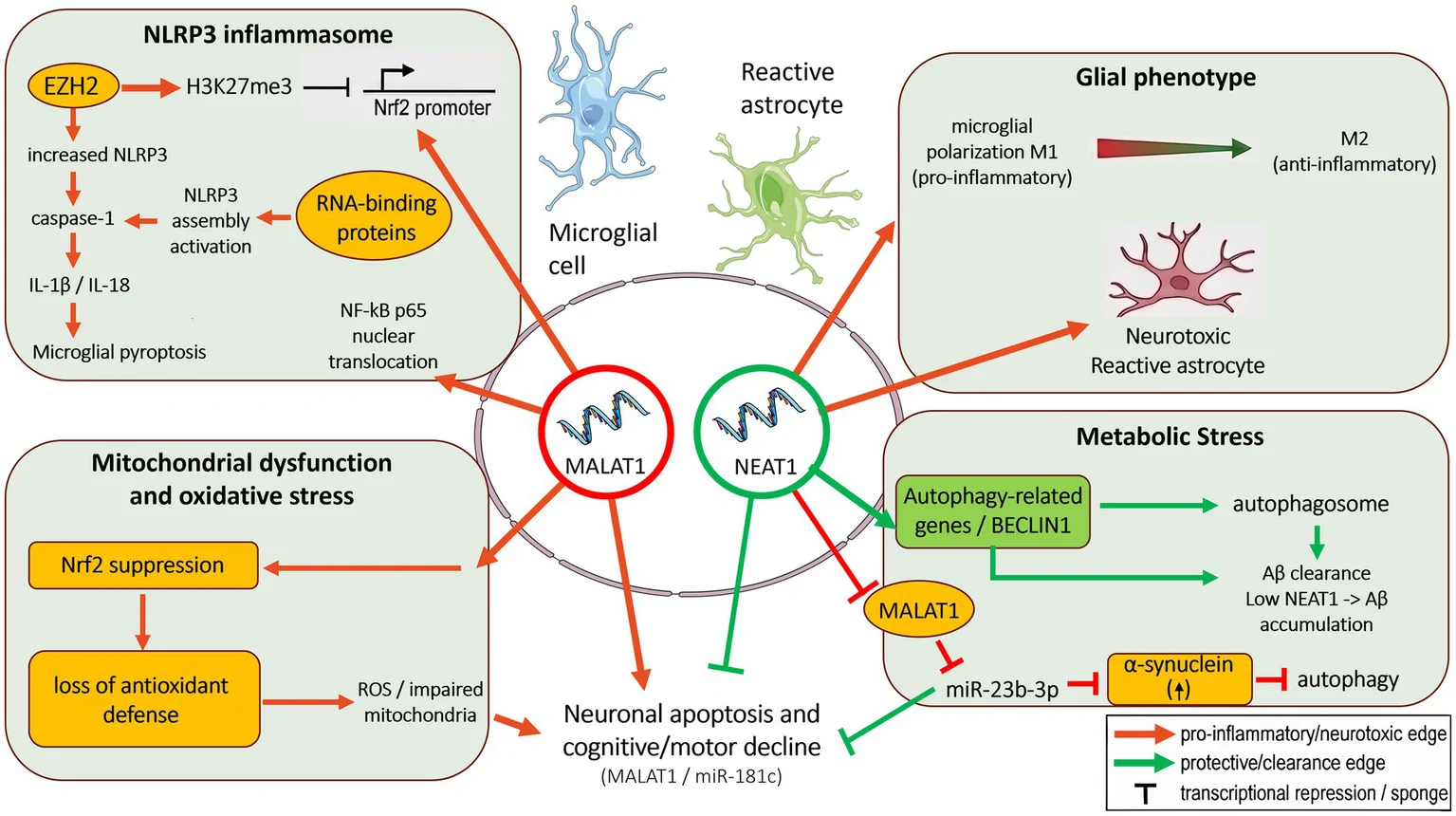
Convergent molecular mechanisms of MALAT1 and NEAT1 in glial neuroinflammation. Within microglia and astrocytes, MALAT1 and NEAT1 act as central hubs driving four interconnected pathological programs. (i) NLRP3 inflammasome activation: MALAT1 promotes NF-κB p65 nuclear translocation and, together with RNA-binding proteins, supports NLRP3 assembly, leading to caspase-1 activation, IL-1β/IL-18 release, and microglial pyroptosis; MALAT1 also recruits EZH2 to deposit the repressive histone mark H3K27me3 at the Nrf2 promoter, silencing its transcription. (ii) Mitochondrial dysfunction and oxidative stress: Nrf2 suppression removes antioxidant defenses, increasing ROS production and mitochondrial impairment. (iii) Glial phenotype switching: MALAT1 upregulation promotes a pro-inflammatory microglial phenotype and drives astrocytes toward a neurotoxic reactive state. (iv) Metabolic stress and impaired clearance: NEAT1 induces autophagy-related genes, including BECLIN1, supporting autophagosome formation and amyloid-β (Aβ) clearance; low NEAT1 levels are associated with Aβ accumulation. Conversely, MALAT1 represses miR-23b-3p, which normally suppresses α-synuclein; this derepression raises α-synuclein levels and impairs autophagy. These converging pathways culminate in neuronal apoptosis and cognitive/motor decline, partly mediated by the MALAT1/miR-181c axis. Red arrows: Pro-inflammatory/neurotoxic effects; green arrows: Protective/clearance effects; flat-headed bars: Transcriptional repression or miRNA sponging. Cell illustrations were created using Servier Medical Art (https://smart.servier.com/); Servier Medical Art is licensed under CC BY 4.0 (https://creativecommons.org/licenses/by/4.0/). Aβ, amyloid-beta; BECLIN1, Beclin 1; ceRNA, competing endogenous RNA; EZH2, enhancer of zeste homolog 2; H3K27me3, histone H3 lysine 27 trimethylation; IL-1β/IL-18, interleukin-1β/18; lncRNA, long non-coding RNA; miRNA, microRNA; NF-κB, nuclear factor kappa-light-chain-enhancer of activated B cells; NLRP3, NOD-, LRR- and pyrin domain-containing protein 3; Nrf2, nuclear factor erythroid 2–related factor 2; ROS, reactive oxygen species.

While BACE1-AS has been historically characterized in neuronal populations, recent landmark studies have pivoted the focus toward its critical regulatory roles in glial cells, directly linking it to neuroinflammation and astrocytic amyloidosis. For instance, Sil et al. demonstrated that exposing human primary astrocytes to pathological insults triggers the activation and translocation of hypoxia-inducible factor 1-alpha (HIF-1*α*), which directly binds to the BACE1-AS promoter. The resulting BACE1-AS/BACE1 RNA complex stabilizes the protease mRNA, thereby stimulating autonomous A\β production within the astrocytic population (Sil et al., 2020). Consistently, emerging evidence in models of toxic-induced neurodegeneration shows that stress-induced upregulation of BACE1-AS in astrocytes concurrently drives amyloid pathways and a robust inflammatory response. Crucially, a gene-silencing approach targeted at BACE1-AS was capable of reversing this phenotype, significantly downregulating the expression of key pro-inflammatory cytokines, including TNF-α, IL-1β, and IL-6 (Kumar et al., 2024). These findings collectively highlight BACE1-AS as a multifunctional glial hub that bridges metabolic stress, non-neuronal aggregate processing, and sustained neuroinflammation. Furthermore, it is known that the expression of the enzyme BACE1 is modulated by miR-29c-3p, which alleviates Aβ-induced apoptosis and neurotoxicity (Cao Y. et al., 2021; Wang et al., 2023). LncRNA PRR34-AS1 negatively modulates miR-29c-3p through a ceRNA mechanism, inducing inflammation and microglial apoptosis. The study conducted by Cheng X. confirmed the serum increase in lncRNA PRR34-AS1 in AD patients compared to healthy controls. PRR34-AS1 expression is also increased in human (HMC3) and murine (BV2) microglial cell lines in response to treatment with Aβ25-35 in a dose-dependent manner. Instead, its silencing inhibited the release of pro-inflammatory factors and improved cell viability (Cheng et al., 2025). However, further studies would be needed to validate the action of the PRR34-AS1/miR-29c-3p axis in animal models and on larger clinical samples. A particular class of lncRNA discovered recently is that of SINEUPs, which act as post-transcriptional regulators. SINEUPs are able to increase the expression of target mRNAs (Zucchelli et al., 2015). They have a binding domain that confers specificity to the SINEUP and binds to the 5’ UTR region of the target mRNA. The effector domain is the element that controls the increase in mRNA translation (Zucchelli et al., 2015). Espinoza et al. tested the efficacy of miniSINEUP-GDNF on mouse astrocytes, demonstrating a significant increase in Glial cell line-derived neurotrophic factor (GDNF) protein levels. Since GDNF is a neurotrophic factor that contributes to the neuroprotection of DAergic terminals, the use of SINEUP-GDNF may represent an innovative strategy to increase extracellular GDNF secretion as a therapeutic treatment for PD (Espinoza et al., 2020). In summary, recent data indicate that dysregulation of lncRNA is closely associated with pathological outcomes. The functional effects and related lncRNAs involved are summarized in Figure 3.

## 10 Conclusion

Identifying the complex mechanisms by which lncRNAs act in the pathogenesis of NDs offers new perspectives on their role as modulators of glial cells toward an inflammatory or protective phenotype (Zohar et al., 2023). Furthermore, they show significant potential for the development of diagnostic biomarkers in patients’ blood for the differential diagnosis of disease. For instance, the use of MIAT and NEAT1 lncRNAs related to the development of AD (Ou et al., 2021), as well as MAGI2-AS3 to detect the severity of this disease (Zhang and Wang, 2021). Recent transcriptomic analyses conducted on postmortem human brains have identified RN7SL1 as a key lncRNA for the diagnosis of AD (Liu et al., 2024). Its expression is, in fact, altered and increases as the disease progresses. In particular, it has been demonstrated that RN7SL1 modulates the microglial response to Aβ oligomers, while its knockdown significantly reduces the production of pro-inflammatory cytokines (Liu et al., 2024).

Moreover, lncRNAs may be used as novel therapeutic targets. The knockdown of specific lncRNAs (e.g., ANRIL, MAGI2-AS3, SNHG14, HOXA11-AS, DLX6-AS1) has shown neuroprotective effects, reducing neuronal apoptosis and neuroinflammation in disease models, (Liu et al., 2022; Cao H. et al., 2021; Zhang et al., 2019; Yang et al., 2021). Another important therapeutic strategy is the potential application of SINEUPs, which have demonstrated effectiveness in enhancing the expression of neuroprotective agents in both *in vitro* and *in vivo* studies. One advantage of SINEUPs over other gene therapy platforms is that they only act when the target mRNA is already expressed. Therefore, through selective enhancement of protein translation, they can therefore exert neuroprotective effects (Espinoza et al., 2020).

However, the studies selected in this review have some limitations. First, they are often performed on small sample size, hence future in vitro/in vivo studies should be conducted on larger cohorts to fully confirm the clinical role of lncRNAs (Gil-Jaramillo et al., 2023; Mosquera-Heredia et al., 2024; Song et al., 2021). Second, many studies focused on specific brain regions, such as the prefrontal cortex or hippocampus, suggesting the need for more comprehensive research involving different brain regions to validate the data (Xie et al., 2024; Schröder et al., 2025). Third, numerous results come from bioinformatic analyses and studies on in vitro models, which require confirmation through relevant in vivo and better understanding of the molecular mechanisms involved (Xie et al., 2024; Gao et al., 2021). Fourth, a critical knowledge gap identified in this systematic review concerns the near-total absence of literature investigating the regulatory roles of lncRNAs within the oligodendrocyte lineage—specifically mature oligodendrocytes and oligodendrocyte precursor cells (OPCs)—in relation to cognitive decline. Although these cell types are acknowledged as active surveillance players in the brain parenchyma and key modulators of synaptic plasticity, the existing literature remains heavily biased toward microglial and astrocytic populations. Deconstructing how non-coding transcripts control oligodendrocyte dysfunction and remyelination failure represents an essential and unmapped frontier for future neurodegenerative research. A further limitation is that computational genomics studies based on text mining are inherently affected by a literature bias. This bias arises manifests itself in the overrepresentation of highly investigated genes, to the detriment of lesser-known or neglected molecules. Consequently, the analyzed sequence sets tend not to be exhaustive, omitting crucial regulatory sequences that are only sporadically mentioned in the scientific literature or have been studied exclusively in specific animal models (El Idrissi et al., 2021). Finally, a critical gap is the scarcity of sex-disaggregated data; as many studies utilized single-sex models or failed to report sex-specific differences, integrating sex as a biological variable remains essential for the precision of future lncRNA-based therapies, given the known sexual dimorphism in neuroinflammation. In line with efforts to actively modulate the glial inflammatory microenvironment other formulations have been designed to improve the glial inflammatory microenvironment. For example, a non-peptide analog of angiotensin 1–7 (AVE0991) has been shown to improve cognitive function and inhibit the NLRP3 inflammasome in a mouse model of AD (Duan et al., 2021). Multifunctional delivery systems are also being developed, such as RVG-DDQ/PDP@siBACE1 nanoparticles. These can cross the BBB and actively modulate the inflammatory microenvironment by reducing reactive oxygen species and regulating the microglial phenotype and Aβ production (Ke et al., 2024). Therefore, the efforts to develop more effective delivery systems will be more and crucial in the following years for transforming lncRNAs into targeted therapeutic tools against inflammation and neurodegeneration.

## Data Availability

The original contributions presented in the study are included in the article/supplementary material, further inquiries can be directed to the corresponding author.

## Glossary

6-OHDA: 6-hydroxydopamine
Aβ: amyloid-beta
AD: Alzheimer’s disease
ALS: amyotrophic lateral sclerosis
*α*-Syn: alpha-synuclein
APP: amyloid precursor protein
ATG: autophagy-related genes
ATP: adenosine triphosphate
BACE1: beta-site APP-cleaving enzyme 1
BBB: blood–brain barrier
ceRNA: competing endogenous RNA
circRNA: circular RNA
CNS: central nervous system
COX2: cyclooxygenase-2
CTSB/CTSD: cathepsin B/D
DAergic: dopaminergic
DGCR8: DiGeorge syndrome Critical Region gene 8
EVs: extracellular vesicles
EZH2: enhancer of zeste homolog 2
FSTL1: Follistatin-like 1
GCN5L2: General Control of Amino Acid Synthesis 5-like 2
GDNF: glial cell line-derived neurotrophic factor
GWAS: Genome-Wide Association Studies
H3K27me3: methylation of histone H3 lysine 27
HSPA5: heat shock protein family A member 5
IL-1β/IL-6: interleukin-1 beta/6
iNOS: inducible nitric oxide synthase
iPSC: induced pluripotent stem cells
LAMP1: lysosomal-associated membrane protein 1
lncRNAs: long non-coding RNAs
LPS: lipopolysaccharide
miRNA: microRNA
MPP+: 1-methyl-4-phenylpyridinium
MPTP: 1-methyl-4-phenyl-1,2,3,6-tetrahydropyridine
mRNA: messenger RNA
NAD+: nicotinammide adenina dinucleotide
NDs: neurodegenerative diseases
NF-κB: nuclear factor kappa-light-chain-enhancer of activated B cells
NLRP3: nod-like receptor protein 3
NMNAT2: nicotinamide nucleotide adenylyltransferase 2
Npas3: neuronal PAS domain protein 3
Nrf2: nuclear factor erythroid 2-related factor
NRP1: neuropilin-1
OPCs: oligodendrocyte precursor cells
PA: palmitic acid
PBMCs: peripheral blood mononuclear cells
PD: Parkinson’s disease
PGC-1α: peroxisome-proliferator-activated receptor-gamma coactivator 1-alpha
PRC2: polycomb repressive complex 2
PRISMA: Preferred Reporting Items for Systematic Reviews and Meta-analyses
PRRs: pattern recognition receptors
RNA pol: RNA polymerase
ROS: reactive oxygen species
siRNA: small interfering RNA
TLRs: toll-like receptors
TNFα: tumor necrosis factor alpha
UTRs: untranslated regions
