# Correction: Long non-coding RNAs in glial cells: key drivers of neuroinflammation in cognitive disorders

**DOI:** 10.3389/fnagi.2026.1972571

**Published:** 2026-09-03

**Authors:** Chiara Malatino, Ivana Raffaele, Angelo Quartarone, Gabriele Raciti, Ivan Anchesi, Nunzio Iraci, Maria Cristina De Cola

**Affiliations:** 1 Policlinico Universitario G. Martino di Messina – UOC Patologia Clinica, Messina, Italy; 2 IRCCS Centro Neurolesi “Bonino-Pulejo”, Via Provinciale Palermo, Contrada Casazza, Messina, Italy; 3 Department of Biomedical and Biotechnological Sciences, University of Catania, Catania, Italy

**Keywords:** cognitive disorders, glia, long non-coding RNAs, neurodegenerative diseases, neuroinflammation, neuroprotection

In the published article, **Figure 1** and **Figure 2** were wrongly inverted; the PRISMA flow diagram appeared under the caption of **Figure 1** (“ncRNAs modulate glial cells along a continuum of functional states...”), while the schematic of the glial functional state continuum appeared under the caption of **Figure 2** (“PRISMA flow diagram describing the study selection process.”). The captions themselves were correctly worded and correctly positioned with respect to their in-text citations; only the two images have been exchanged. The order has now been corrected.

In the published article, in the **Funding** section, the funder “European Union – NextGenerationEU, PNRR M6C2 – Investment 2.1, project code PNRR-MCNT1-2023-12378447 (CUP: I43C24000190006)” was erroneously omitted. The corrected **Funding** statement appears below.

“The author(s) declared that financial support was received for this work and/or its publication. This study was supported by the European Union—Next Generation EU (project code PNRR-MCNT1-2023-12378447). This study was also funded by the European Union—Next Generation EU—PNRR M6C2—Investment 2.1 (project code PNRR-MCNT1-2023-12378447—CUP: I43C24000190006).”

The original version of this article has been updated.

